# Full-length transcriptome of *Oocystis borgei* under stress condition

**DOI:** 10.3389/fgene.2023.1255595

**Published:** 2023-10-17

**Authors:** Chengcheng Deng, Jiajia Ren, Ting Hong, Yang Liu, Feng Li, Yulei Zhang, Changling Li, Zhongdian Dong, Xianghu Huang, Ning Zhang

**Affiliations:** ^1^ Lab of Algae Resource Development and Aquaculture Environment Ecological Restoration, Fisheries College, Guangdong Ocean University, Zhanjiang, China; ^2^ Key Laboratory of Aquaculture in South China Sea for Aquatic Economic Animal of Guangdong Higher Education Institutes, Fisheries College, Guangdong Ocean University, Zhanjiang, China

**Keywords:** *Oocystis borgei*, microalga, full-length transcriptome, lncRNA, annotation

## Introduction

*Oocystis borgei* is a green microalga that was originally isolated from a subtropical *L. vannamei* pond, and it exhibits remarkable characteristics of population stability and environmental adaptiveness (Huang et al., 2002a). Unlike other microalgal species that can cause red tides and mass mortality due to rapid, short-term proliferation, the *Oocystis borgei* cells proliferate slowly and maintain relatively stable water conditions (Huang et al., 2002b). In aquaculture settings, *O. borgei* forms a stable niche, that effectively competes harmful microalgae and aids in the absorption of dissolved nitrogen, while inhibiting the growth of pathogenic *Vibrio*, thereby contributing to the health of *Litopenaeus vannamei* (Huang et al., 2005; Li et al., 2010; Huang et al., 2012; Liu et al., 2018; Liu et al., 2020; Huang and Li, 2021; Wang et al., 2022; Chen et al., 2023). However, the lack of theoretical research on *O. borgei* hampers our understanding of its regulation of water quality, which limits further advancement and utilization of this microalga. The biological characteristics of a species are determined by its hereditary factors, and acquiring information on the genome and functional genes of *O. borgei* is crucial to elucidate its biological characteristics. Unfortunately, the genomic information of *O. borgei* remains elusive, which impedes comprehensive functional studies.

Functional genes located in the nucleus undergo transcription into RNA, which then carries the genetic information into the cytoplasm and facilitates the synthesis of functional proteins on the ribosome. Serving as a link between genes and proteins within a species, the transcriptome provides the foundation for investigating gene structure and function. Transcriptome sequencing technology can map the selective profile of expression of the functional genes in the current state of a sample and obtain mRNA sequence characteristics and the patterns of expression of functional genes (Hrdlickova et al., 2017). However, traditional transcriptome sequencing technology based on second generation high-throughput sequencing platforms often possesses limitations in accurately obtaining or assembling complete transcripts and identifying gene isoforms or homologous genes due to the read length constraints, which results in incomplete transcript information and reduced accuracy in the analysis of gene expression and alternative splicing (Wang et al., 2009; Liao et al., 2015).

In contrast, full-length transcriptome sequencing based on the Pacific platform provides full-length transcript information without the need for cDNA disruption or splicing since it directly sequences the target RNA. This generates extremely long reads averaged 15 kb using RACE technology for reverse transcription. This approach yields highly accurate sequencing (99.9%) and enables a comprehensive understanding of all the RNA molecules in an organism, including all the transcripts and alternative splicing isoforms. The scope of application of full-length transcriptome sequencing is widespread and includes studying the regulation of gene expression, discovering new genes, and identifying alternative splicing isoforms and more (Liao et al., 2015; Hu et al., 2020).

To obtain the functional gene information and provide basis for future studies, the full-length transcriptome of *O. borgei* derived from 11 different culture conditions was analyzed using PacBio single molecule real-time (SMRT) sequencing. Moreover, annotating the gene set of *O. borgei* could enhance the annotation of the entire genome, which would contribute to a better understanding of the complexity of the genome and serve as a reference sequence for gene functional studies.

## Materials and methods

### Cultivation of *O. borgei*

*O. borgei* was obtained from the Lab of Algae Resource Development and Aquaculture Environment Ecological Restoration at Guangdong Ocean University, Zhanjiang, China. The stock cultures of *O. borgei* were pre-cultivated indoors using modified f/2 medium (Liu et al., 2020) for 2 weeks. The culture conditions were as follows: artificial seawater salinity of 30 parts per thousand (PPT), temperature maintained at 25°C, light intensity set at 120 μmol·m^-2^·s^-1^, and a light cycle of 12 h light/12 h dark. Nitrogen and carbon sources were provided using sodium nitrate (NaNO_3_) at a concentration of 80 mg·L^-1^ and NaHCO_3_ at a concentration of 173.5 mg·L^-1^, respectively. Following pre-cultivation, the microalgae were concentrated through gravity precipitation and subsequently washed with 1 x phosphate buffer saline (PBS) via centrifugation (6,000 g for 10 min). The stock cultures of *O. borgei* were then inoculated with approximately 5 × 10^5^ cells L^-l^ (OD_680_ = 0.137) and cultured in 500 mL conical flasks that contained 450 mL of the respective medium. The cultures were maintained in a constant temperature light incubator (Life Apparatus, China). To sufficiently gather the full-length transcriptome of *O. borgei*, three abiotic factors, namely, temperature, light intensity, and salinity, along with two nutritional factors, the concentrations of nitrogen and carbon, were selected for manipulation (Table 1). The temperature and light intensity were adjusted using the parameters of the constant temperature light incubator, while the control over salinity and the concentrations of nitrogen and carbon was achieved by modifying the concentrations of NaCl, NaNO_3_, and NaHCO_3_ in the medium, respectively. After 7 days of cultivation with five daily shaking cycles, the microalgal samples were enriched through centrifugation at 12000 *g* for 15 min and subsequently ground using liquid nitrogen for total RNA extraction (Supplementary Figure S1).

**TABLE 1:** Cultivation of *Oocystis borgei* under different stress conditions.

Factor	Temperature (°C)	Light intensity (μmol·m^-2^·s^-1^)	Salinity (PPT)	NaNO_3_ (mg·L^-1^)	NaHCO_3_ (mg·L^-1^)
Normal	**25**	**120**	**30**	**80**	**173.5**
High temperature	**35**	120	30	80	173.5
Low temperature	**15**	120	30	80	173.5
High light intensity	25	**400**	30	80	173.5
Low light intensity	25	**32**	30	80	173.5
High salinity	25	120	**50**	80	173.5
Low salinity	25	120	**10**	80	173.5
High Nitrogen concentration	25	120	30	**5236**	173.5
Nitrogen Free	25	120	30	**0**	173.5
High Carbon concentration	25	120	30	80	**867.5**
Carbon-Free	25	120	30	80	**0**

The bold values indicate the culture conditions of Oocystis borgei in the control group and the changed environmental factors in each treatment group.

### Total RNA preparation and SMRT sequencing

The total RNA was extracted using an RNAprep Pure Plant Plus Kit (TIANGEN, China) following the manufacturer’s instructions. The integrity of the total RNA was assessed using 1% agarose gel electrophoresis and the Agilent 2100 Bioanalyzer (Agilent Technologies, USA) (Supplementary Figure S2). The purity and concentration of the total RNA were determined using the Nanodrop micro-spectrophotometer (Thermo Fisher Scientific, USA) (Supplementary Table S1). Subsequently, 0.5 μg of the total RNA from each sample was pooled into a single pool, which was sent to Gene *Denovo* Biotechnology Co. (Guangzhou, China) for quality testing, cDNA library construction, and sequencing. Messenger RNA (mRNA) was enriched from the mixed total RNA using Oligo (dT) magnetic beads and reverse transcribed into cDNA using the Clontech SMARTer PCR cDNA Synthesis Kit. The PCR cycle was optimized to determine the optimal amplification cycle number for the downstream large-scale PCR reactions. The optimized cycle number was then used to generate double-stranded cDNA. Moreover, size selection >5 kb was conducted using the BluePippin™ Size-Selection System, and this selected cDNA was mixed equally with the cDNA that had not been selected for size. Subsequently, large-scale PCR was performed to construct the SMRT bell library. The cDNAs were subjected to DNA damage repair, end repair, and ligation with sequencing adapters. The SMRT bell template was annealed to the sequencing primer, bound to the polymerase, and sequenced on the PacBio Sequel II platform.

### Transcriptome assembly and data analysis

The raw data were analyzed using an isoform sequencing (Iso-Seq) pipeline supported by Pacific Biosciences (Gordon et al., 2015). Firstly, high-quality circular consensus sequence (CCS) reads were extracted from the subreads BAM file, and the integrity of transcripts was assessed based on the presence of 5′primer, 3′primer, and poly-A sequences using isoseq3. Sequences that contained all three structures were considered to be full-length (FL) reads. After the primers, barcodes, poly-A tails, and concatemers of full passes had been removed, the full-length non-chimeric (FLNC) reads were obtained. These FLNC reads were then clustered to generate complete isoforms, and similar reads were hierarchically clustered using Minimap2 to obtain consensus sequences. The quiver algorithm was subsequently applied to further refine the consensus sequences. Only high-quality isoforms with a prediction accuracy of ≥0.99 were retained for subsequent analysis. The CD-HIT program was used to remove redundancy with a threshold identity of 0.99. The quality of the final isoforms were assessed using the Benchmarking Universal Single-Copy Orthologs (BUSCO) with eukaryota_odb9 (Simão et al., 2015).

The final isoforms were aligned to the NCBI non-redundant protein (Nr) database (http://www.ncbi.nlm.nih.gov), Swiss-Prot protein database (http://www.expasy.ch/sprot), Kyoto Encyclopedia of Genes and Genomes (KEGG) database (http://www.genome.jp/kegg), and Clusters of Orthologous Groups (COG)/Eukaryotic Orthologous Groups (KOG) database (http://www.ncbi.nlm.nih.gov/COG) using the BLASTx program (http://www.ncbi.nlm.nih.gov/BLAST/). A threshold value of 1e^−5^ was utilized to assess the sequence similarity with the genes from other species. To further classify the molecular functions of *O. borgei* transcripts, Gene Ontology (GO) annotation was performed using the Blast2GO software (Conesa et al., 2005) based on the Nr annotation results of the isoforms. Isoforms that ranked among the top 20 highest scores and with a length of at least 33 High-scoring Segment Pair (HSP) hits were selected for Blast2GO analysis. Subsequently, the functional classification of the isoforms was conducted using the Web Gene Ontology Annotation Plot (WEGO) software (Ye et al., 2006).

Open reading frames (ORFs), coding sequences (CDS), protein sequences, and untranslated regions (UTRs) of the isoforms were detected using a combination of BLAST and ANGEL software (Shimizu et al., 2006). Long non-coding RNAs (lncRNAs) were identified by analyzing full-length transcript sequences without annotations in the four major databases. The Coding-Non-Coding Index (CNCI) software (Version 2) (Sun et al., 2013) and Coding Potential Calculator (CPC) software (Kong et al., 2007) were utilized to predict the coding potential, and those transcripts predicted as “non-coding” by both software were considered to be lncRNAs. Additionally, alternative splicing (AS) events of the transcript isoforms were analyzed using the COding GENome reconstruction Tool (Cogent) (Li et al., 2017) and the SUPPA tool (https://bitbucket.org/regulatorygenomicsupf/suppa) (Alamancos et al., 2015). The protein domains predicted using the Pfam database (Version 26.0) (Finn et al., 2016) and the SMART database (Version 06/08/2012) with the Pfam_Scan program. Transmembrane helices and signal peptides were predicted using the online HMMER2.0 Server (http://www.cbs.dtu.dk/services/TMHMM/) and SignalP 4.1 Server (http://www.cbs.dtu.dk/services/SignalP/), respectively. The NetOGlyc 4.0 Server (http://www.cbs.dtu.dk/services/NetOGlyc/) and ProP 1.0 Server (http://www.cbs.dtu.dk/services/ProP/) were utilized to predict mucin-type GalNAc O-glycosylation sites and arginine and lysine propeptide cleavage sites, respectively. Moreover, isoform protein coding sequences were aligned to the Plant Transcription Factor Database (Plant TFdb) (http://planttfdb.cbi.pku.edu.cn/) using hmmscan to predict transcription factor (TF) families. Finally, microsatellite mining was performed on the transcriptome using MIcroSAtellite (MISA, http://pgrc.ipk-gatersleben.de/misa/). The parameters used for microsatellite identification were set to a minimum of 2–6, 3-5, 4-4, 5-4, and 6-4 repetitions for each repeat unit type. For instance, 2-6 indicates that a dinucleotide repeat type must be repeated at least six times to be considered a microsatellite. If two simple sequence repeats (SSRs) are separated by a distance of <100 bp, they are considered to be a single SSR.

## Results

**FIGURE 1:**
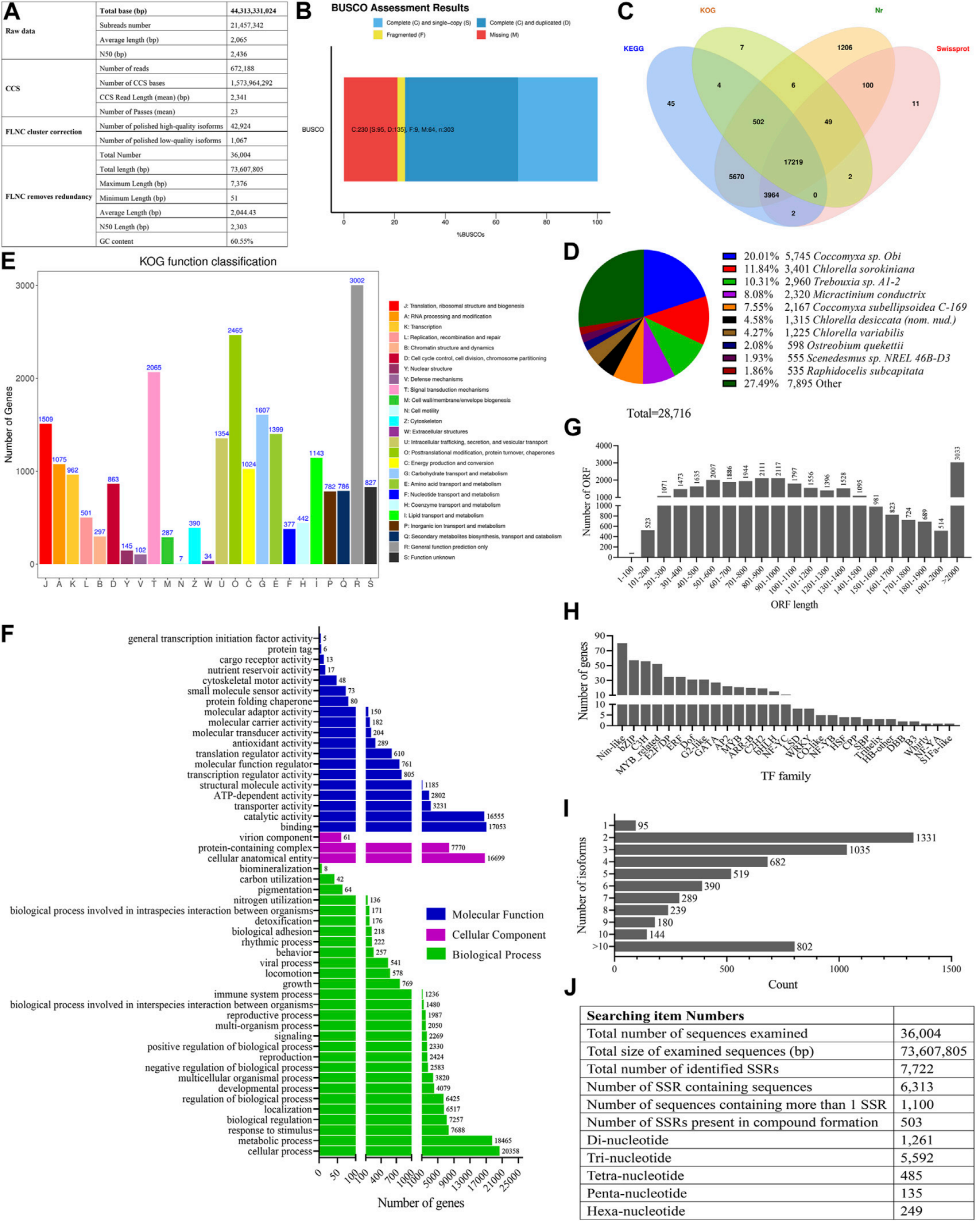
Statistics analysis of full-length transcriptome of *Oocystis borgei*. **(A)** Summary of the full-length transcriptome of *O. borgei*. **(B)** BUSCO integrity assessment of the transcriptome data. **(C)** Venn diagram illustrating the basic gene annotation of *O. borgei*. **(D)** Distribution of homologous species in the *O. borgei* transcripts annotated in the Nr database. **(E)** KOG classification of the *O. borgei* transcripts. The *x*-axis represents subcategories, and the *y*-axis indicates the number of transcripts in each functional cluster. **(F)** GO classification of the *O. borgei* transcripts. The *y*-axis represents GO categories, and the *x*-axis represents the number of transcripts. **(G)** Distribution of the lengths of complete open reading frames (ORFs). **(H)** Distribution of transcription factor (TF) families in the full-length transcriptome of *O. borgei*. **(I)** Overview of alternative splicing (AS) analyses in the full-length transcriptome of *O. borgei*. **(J)** Summary of simple sequence repeats (SSRs) identified in the *O. borgei* transcriptome. BUSCO, Benchmarking Universal Single-Copy Orthologs; GO, Gene Ontology; KOG, eukaryotic orthologous groups.

A total of 21,457,342 raw subreads, which contained 44,313,331,024 base pairs (bp), were obtained from the cDNA library of *O. borgei*, with an average length and N50 of 2,065 bp and 2,436 bp, respectively (Figure 1A). A total of 672,188 CCS reads were generated from the subreads, with an average length of 2,341 bp. Among these reads, 42,924 high-quality isoforms and 1,067 low-quality isoforms (prediction accuracy <0.99) were identified as FLNC reads. After removing the redundancy with CD-HIT, 36,004 FLNCs were obtained in the transcriptome of *O. borgei*, with an average length and N50 of 2,044 bp and 2,303 bp, respectively (Figure 1A). The results of BUSCO analysis indicated that there were 75.91% complete BUSCOs (31.35% single-copy and 44.56% duplicated), 21.12% missing BUSCOs, and 2.97% fragmented BUSCOs (Figure 1B).

The annotation analysis revealed that out of the 36,004 FLNCs, 28,787 transcripts (79.96%) were successfully annotated, with 28,716, 17,789, 27,406, and 21,347 transcripts annotated in the NR, KOG, KEGG, and Swiss-Prot databases, respectively (Figure 1C; assembly and annotated information in Supplementary Tables S2, S3). The species distribution analysis indicated that the aligned transcripts belonged to 351 known species. Among them, the highest proportion of transcripts was distributed in *Coccomyxa* sp. *Obi* (20.01%), followed by *Chlorella sorokiniana* (11.84%), *Trebouxia* sp. *A1-2* (10.31%), and *Micractinium conductrix* (8.08%) (Figure 1D).

The KOG analysis categorized 17,789 transcripts into 25 functional categories. The largest group was “General function prediction only” (3,002 transcripts, 16.88%), followed by “Posttranslational modification, protein turnover, chaperones” (2,465 transcripts, 13.86%) and “Signal transduction mechanisms” (2,065 transcripts, 11.61%). The categories with the fewest transcripts were “Extracellular structures” (34 transcripts, 0.19%) and “Cell motility” (7 transcripts, 0.039%) (Figure 1E). Furthermore, 27,406 transcripts were mapped to 130 KEGG functional pathways. The dominant pathways were “Metabolic pathways” (5,807 transcripts, 21.19%) and “Biosynthesis of secondary metabolites” (3,148 transcripts, 11.49%) (Supplementary Table S4).

In the GO annotation analysis, the transcripts were classified into three main categories, including “Biological processes”, “Cellular components”, and “Molecular functions”. The most enriched groups in the biological process category were “Cellular process” (20,358 transcripts) and “Metabolic process” (18,465 transcripts)". In the cellular component category, “Cellular anatomical entity” (16,699 transcripts) was the most enriched group. In the molecular function category, the transcripts were predominantly involved in “Binding” (17,053 transcripts) and “Catalytic activity” (16,555 transcripts) (Figure 1F).

The gene structure analysis predicted a total of 33,217 ORFs, including 28,903 complete ORFs with sequence lengths that ranged from 102 bp to 6,693 bp. Most of the complete ORFs (25,870 out of 28,903, 89.51%) fell within the range of 101-2,000 bp, which indicated that the isoforms were high-quality (Figure 1G).

Among the 33,217 coding isoforms, the Pfam database identified 25,169 isoforms with 40,959 protein domains, while the SMART database predicted 8,683 isoforms with 16,235 protein domains. The TMHMM analysis predicted that 5,027 isoforms contained transmembrane helices, and SignalP 4.1 Server predicted 2,446 proteins with signal peptide cleavage sites. Furthermore, 18,525 proteins were found to have O-GlcNAc glycosylation sites, and 7,104 proteins had ProP furin cleavage sites (Supplementary Tables S5, S6).

The analysis of TF families revealed that the Nin-like TF family contained the highest number of isoforms (80), followed by the bZIP (57), C3H (56), and MYB-related TF families (52) (Figure 1H). lncRNAs, which play roles in organism growth and stress responses, were predicted using both the CNCI and CPC software. A total of 1,227 FLNC reads were jointly predicted as lncRNAs (Supplementary Table S7). Moreover, 5,724 genes were found to undergo AS, with 1,331 genes that had two isoforms (Figure 1I). Due to the lack of an available *O. borgei* reference genome, the characterization of AS event types and prediction of lncRNA target genes were not conducted in this study.

Among all the isoforms, 6,313 isoforms contained 7,722 simple sequence repeats (SSRs). Of these, 1,100 isoforms contained at least two SSRs, and 503 SSRs were identified as compound formations (Figure 1J). The most abundant type of SSR was the tri-nucleotide repeat, which accounted for 72.42% of all the SSRs (5,592 out of 7,722).

In summary, this study provides a full-length transcriptome of *O*. *borgei* under different stress conditions, which provides data and a theoretical reference for the future development of genetic resources of microalgae and research on the species of *Oocystis*.

## Data Availability

The datasets presented in this study can be found in online repositories. The names of the repository/repositories and accession number(s) can be found in the article/Supplementary Material.
