# Supplementary material for: Full-length transcriptome of Oocystis borgei under stress condition
Source: Front Genet. 2023 Oct 17;14:1255595. doi: 10.3389/fgene.2023.1255595 (PMC10616457; doi:10.3389/fgene.2023.1255595)
Supplement: Supplementary file 3 [file DataSheet1.DOCX]

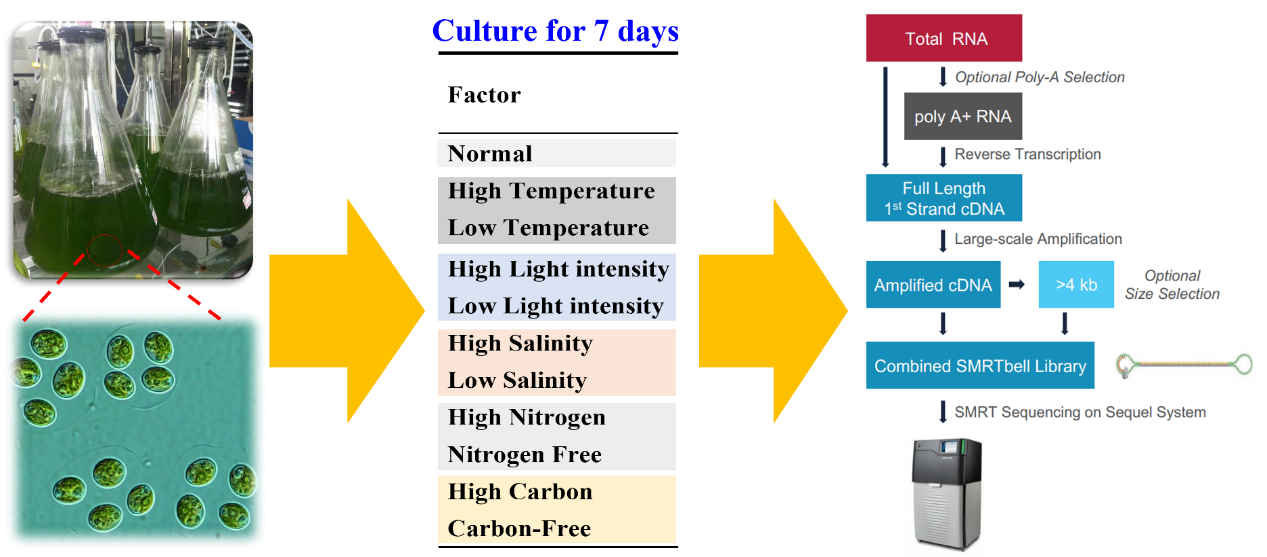


**Figure S1 The experimental design route of this study**


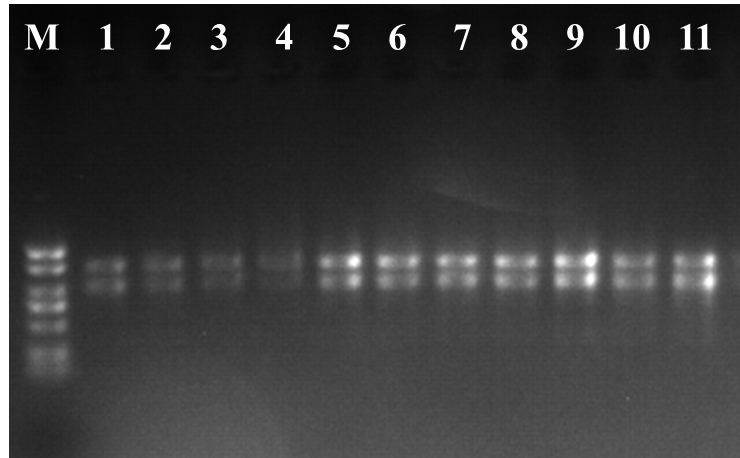


**Figure S2. Results of total RNA sample electrophoresis**

M, DL2000 DNA Marker; 1, Low Light intensity, 2, High Light intensity; 3, Low Temperature; 4, High Temperature; 5, Nitrogen Free; 6, High Nitrogen concentration; 7, Carbon Free; 8, High Carbon concentration; 9, Low Salinity; 10, High Salinity; 11, Normal (Control)
